# The molecular pathology of pathogenic mitochondrial tRNA variants

**DOI:** 10.1002/1873-3468.14049

**Published:** 2021-02-12

**Authors:** Uwe Richter, Robert McFarland, Robert W. Taylor, Sarah J. Pickett

**Affiliations:** ^1^ Wellcome Centre for Mitochondrial Research The Medical School Newcastle University UK; ^2^ Molecular and Integrative Biosciences Research Programme Faculty of Biological and Environmental Sciences University of Helsinki Finland; ^3^ Newcastle University Biosciences Institute Newcastle University UK; ^4^ Newcastle University Translational and Clinical Research Institute Newcastle University UK

**Keywords:** heteroplasmy, m.3243A>G, m.8344A>G, MELAS, MERRF, mitochondrial disease, mitochondrial DNA, mitochondrial tRNA

## Abstract

Mitochondrial diseases are clinically and genetically heterogeneous disorders, caused by pathogenic variants in either the nuclear or mitochondrial genome. This heterogeneity is particularly striking for disease caused by variants in mitochondrial DNA‐encoded tRNA (mt‐tRNA) genes, posing challenges for both the treatment of patients and understanding the molecular pathology. In this review, we consider disease caused by the two most common pathogenic mt‐tRNA variants: m.3243A>G (within *MT‐TL1*, encoding mt‐tRNA^Leu(UUR)^) and m.8344A>G (within *MT‐TK*, encoding mt‐tRNA^Lys^), which together account for the vast majority of all mt‐tRNA‐related disease. We compare and contrast the clinical disease they are associated with, as well as their molecular pathologies, and consider what is known about the likely molecular mechanisms of disease. Finally, we discuss the role of mitochondrial–nuclear crosstalk in the manifestation of mt‐tRNA‐associated disease and how research in this area not only has the potential to uncover molecular mechanisms responsible for the vast clinical heterogeneity associated with these variants but also pave the way to develop treatment options for these devastating diseases.

## Graphical abstract

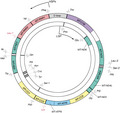

## Abbreviations

**MELAS**, encephalopathy and stroke‐like episodes

**MERRF**, myoclonic epilepsy associated with ragged‐red fibres

**mito‐nuclear**, mitochondrial to nuclear

**mt‐aaRSs**, mitochondrial aminoacyl‐tRNA synthetases

**mt‐tRNA**, mitochondrial tRNA

**OXPHOS**, oxidative phosphorylation

**Fig. 1:**
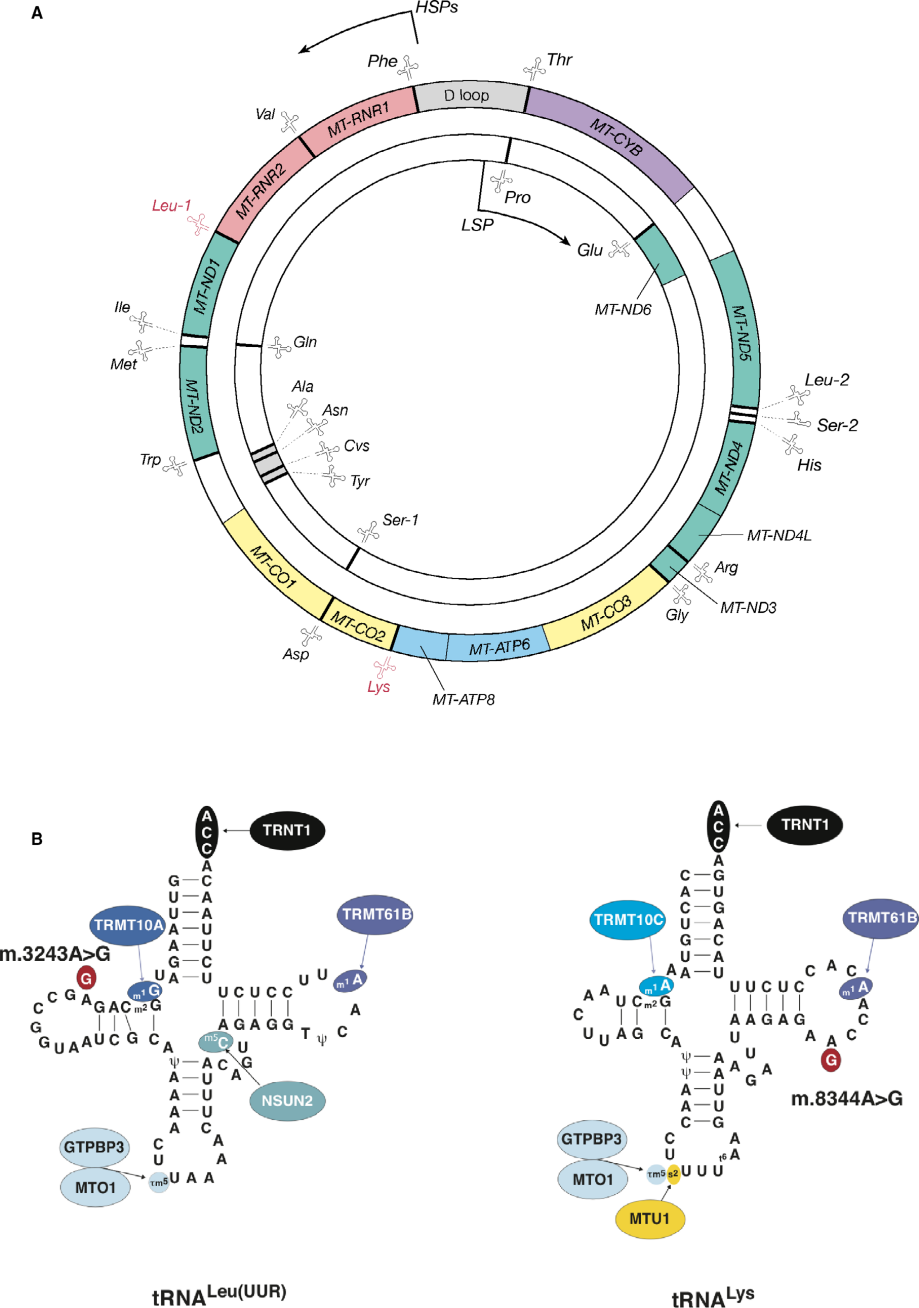
Mitochondrial tRNAs are encoded by the human mitochondrial genome. (A) Schematic representation of the double‐stranded circular human mitochondrial DNA (16.6 kb). The outer circle represents the guanine‐rich heavy strand, which is transcribed from heavy strand promoters 1 and 2 (HSPs), and the inner circle represents the cytosine‐rich light strand, transcribed from the light‐strand promoter (LSP). Thirteen protein‐coding genes encode essential components of the respiratory chain: seven complex I (green; *MT‐ND1* to *MT‐ND6* and *MT‐ND4L*); one complex III (purple; *MT‐CYB*); three complex IV (yellow; *MT‐CO1* to *MT‐CO3*); and two mitochondrial ATP synthase (blue; *MT‐ATP6* and *MT‐ATP8*) subunits. The mitochondrial genome also encodes all of the RNA species required for translation of these polypeptides: *MT‐RNR1* and *MT‐RNR2* encode mitochondrial 12S and 16S rRNAs (red), and 22 genes encoding mt‐tRNA molecules are distributed throughout the genome (grey bars; mt‐tRNA molecules illustrated as clover‐leaf structures with three letter amino acid codes). The two pathogenic variants discussed in this review, m.3243A>G and m.8344A>G, are found within the genes for mt‐tRNA^Leu(UUR)^ (*Leu‐1*) and mt‐tRNA^Lys^ (*Lys*), which are highlighted in red. (B) Schematic depicting the secondary structure of mitochondrial mt‐tRNA^Leu(UUR)^ and mt‐tRNA^Lys^ highlighting the position of m.3243A>G and m.8344A>G (red). Specific RNA modifications and their responsible ‘writer’ enzymes are colour‐coded. Abbreviations for modified nucleosides are as follows: m1A (1‐methyladenosine), m1G (1‐methylguanosine), m2G (N2‐methylguanosine), m5C (5‐methylcytidine), τm5U (5‐taurinomethyluridine), τm5s2U (5‐taurinomethyl‐2‐thiouridine), t6A (N6‐threonylcarbamoyladenosine), Ψ (pseudouridine).

Mitochondria are the principal generators of adenosine triphosphate (ATP) within eukaryotic cells via a process referred to as oxidative phosphorylation (OXPHOS). Mitochondria are double membrane‐bound organelles that are uniquely controlled by their own multicopy genome and that of the nucleus. The human mitochondrial DNA (mtDNA) is a maternally inherited double‐stranded circular genome of 16 569 bp. The overwhelming majority of mitochondrial proteins are encoded by the nuclear genome [1], but the mtDNA encodes 13 polypeptides, each of which forms a core subunit in one of the OXPHOS complexes, with the exception of complex II, which is entirely nuclear‐encoded (Fig. 1A) [2, 3]. To translate these 13 mtDNA‐encoded proteins, mitochondria contain their own set of ribosomes; the proteins for the mitochondrial translation machinery are nuclear‐encoded, translated in the cytoplasm and transported into the mitochondrial matrix, but all of the essential RNA species (16S and 12S ribosomal RNAs and 22 mt‐tRNAs) are mtDNA‐encoded [4]. The dual genetic origin of mitochondrial ribosomes and the respiratory chain imposes a unique challenge for human cells, the need to tightly coordinate the expression of the mitochondrial genome with the nucleo‐cytoplasmic gene expression machinery. Failure to coordinate these processes results in stress and dysfunction, triggering surveillance mechanisms to protect the cell from disruptions in mitochondria. However, chronic perturbations of the system will ultimately lead to human disease [5].

Pathogenic variants in mitochondrial tRNAs cause a wide range of disease phenotypes, but high‐energy‐consuming tissues such as those forming the neuromuscular and nervous systems are particularly vulnerable. As outlined above, mt‐tRNAs are required for mitochondrial protein biosynthesis. Establishing a fully functional tRNA molecule (cytoplasmic or mitochondrial) requires a multitude of post‐ and cotranslational modification steps, including 5′ and 3′ processing, CAA addition and aminoacylation, as well as a myriad of chemical RNA base modifications. tRNA‐modifying enzymes (writer enzymes) have been reported to account for more than a hundred distinct post‐transcriptional modifications on both the ribonucleotide purine/pyrimidine bases and the sugar backbone. The mt‐tRNA‐modifying enzymes and the consequential mt‐tRNA modifications (Fig. 1B) constitute another level of structural information ancillary to the primary structure for folding and recognition, and have both been associated with human pathologies [].

Throughout evolution, mammalian mt‐tRNA genes have also accumulated mutations at significantly higher rate compared with their cytoplasmic counterparts, and as a result, human mt‐tRNAs are structurally condensed and fragile. It has been suggested that this reduced structural complexity in mammalian mt‐tRNAs is compensated by primary sequence‐independent induced‐fit adaptation of and to the cognate mitochondrial aminoacyl‐tRNA synthetases (mt‐aaRSs) [7]. This interaction has potentially important implications for our understanding of mt‐tRNA mutation disease and pathology. In line with this evolutionary trend, the majority of disease‐causing mtDNA mutations in humans are mapped to mt‐tRNA genes. However, the highly polymorphic nature of mt‐tRNA genes hampers the characterisation of novel substitutions found in patients as pathogenic. Also, genetic heterogeneity between cells and tissues seems to be highly relevant for diseases caused by mt‐tRNA mutations and a better understanding of the very complex genotype–phenotype relationship in this group of disorders is of the greatest importance [8, 9].

As exemplars of mitochondrial disease caused by defects in mt‐tRNA molecules, we consider the two most common pathogenic mt‐tRNA variants, namely m.3243A>G (within *MT‐TL1*, encoding mt‐tRNA^Leu(UUR)^) and m.8344A>G (within *MT‐TK,* encoding mt‐tRNA^Lys^), which together account for ~ 85% of all mt‐tRNA‐related mitochondrial disease [10]. They both pose significant challenges for clinical management and genetic counselling as their phenotypic spectra are highly heterogeneous, disease burden varies widely between patients, and treatment options are limited. This complexity is linked with the multicopy nature of mtDNA, which permits the existence of more than one species of mtDNA molecule within a cell, a status known as heteroplasmy (Box 1). Both m.3243A>G and m.8344A>G are heteroplasmic, and the proportion of the disease‐associated allele can vary from 0% to near 100%. Maternal transmission of these heteroplasmic variants through the mitochondrial bottleneck in oogenesis results in variable levels of the pathogenic allele in the offspring of female carriers, contributing to this heterogeneity and highlighting the importance of providing reproductive options for female carriers (reviewed in Ref. [11]). Understanding the molecular pathogenesis of these variants and how they cause such varied disease is critically important for the clinical management of patients, but there is much we currently do not know.

Box 1 **Heteroplasmy**

**A: Intracellular variability**. The multicopy nature of the mitochondrial genome gives rise to heteroplasmy; the coexistence of more than one species of mtDNA molecule within the same cell. Consequently, the proportion of variant alleles within cells can vary from 0% to 100%. Cells at the extremes, containing only one mtDNA species, are termed homoplasmic. Cells that contain a mixture of mtDNA species are termed heteroplasmic. Both m.3243A>G and m.8344A>G are heteroplasmic mtDNA variants, as depicted by the presence of both wild‐type (blue) and variant (red) alleles within the same cell.

**B: Schematic representation of skeletal muscle tissue showing variability in heteroplasmy between fibres**. Cells within tissues show mosaicism; the majority of cells will be heteroplasmic but it is possible for some cells to become homoplasmic, or near homoplasmic, for either wild‐type or variant alleles. Both random segregation of mitochondria into daughter cells during cell division and clonal expansion (due to either random or selective mtDNA replication) contribute to this heterogeneity. The underlying distribution of heteroplasmy within the tissue can be assessed using quantitative, single‐cell assessments.

**C: Levels of m.3243A>G vary between tissues within an individual**. Blood, urine and skeletal muscle samples are frequently taken to assess carrier status and heteroplasmy for pathogenic mtDNA variants as they are relatively easily accessible. These measurements represent homogenate tissue levels, and they can vary from tissue to tissue. For m.3243A>G, mitotic tissues, such as blood, show an age‐related decline in levels of the pathogenic allele and levels in urine are highly variable, whereas levels are more stable in muscle. In patients with the m.8344A>G variant, levels of the pathogenic allele are uniformly distributed across all tissues.

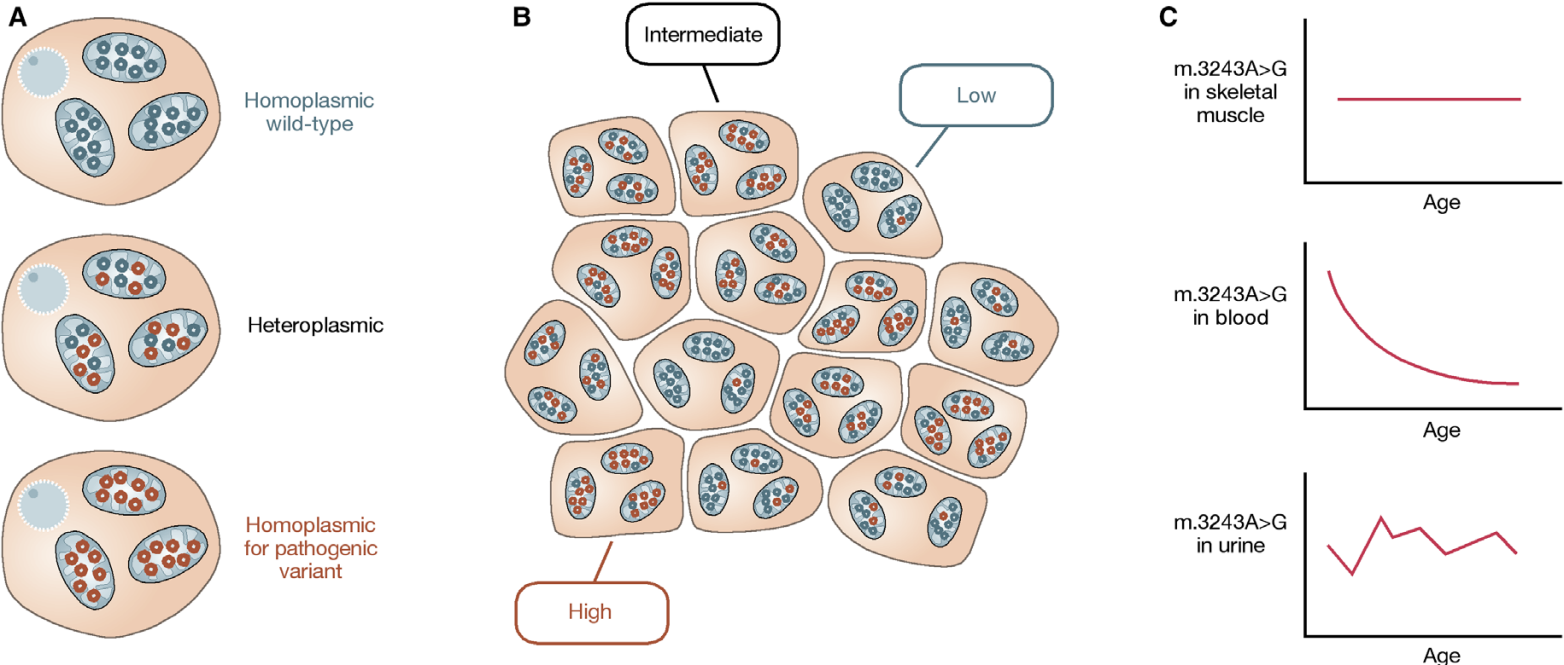

In this review, we outline the phenotypic spectra associated with both m.3243A>G and m.8344A>G and the complexities involved in assessing the effect of variant allele heteroplasmy level on disease burden. Although both variants affect the translation of mtDNA‐encoded proteins and consequently impact OXPHOS function, their molecular pathology and the role of the nucleus in the cellular response to their presence, such as the mitochondrial integrated stress response (ISR^mt^), are not fully understood. We also discuss the advantages of using patient‐derived tissue to study the underlying pathogenic molecular mechanisms and cellular responses and consider how hypothesis‐driven and knowledge‐discovery approaches can be combined to identify factors involved in the vast phenotypic heterogeneity of mt‐tRNA‐related disease, with the ultimate aim of improving patient care.

## Variability in disease burden and progression

### Clinical phenotypes of m.3243A>G

The heteroplasmic m.3243A>G variant was first identified as pathogenic 30 years ago, within a cohort of patients suffering from a maternally inherited syndrome characterised by mitochondrial myopathy, encephalopathy and stroke‐like episodes (MELAS) [12, 13, 14]. Aetiologically distinct from vascular strokes, the stroke‐like episodes associated with MELAS are acute or subacute metabolic crises resulting in brain lesions that often cross anatomical, vascular boundaries and frequently occur before the age of 40 years [15]. Although it has been estimated that 80% of MELAS cases are caused by m.3243A>G [16, 17, 18], rarer mtDNA variants have also been associated with this neurometabolic syndrome, including the m.3271T>C and m.3291T>C *MT‐TL1* variants [16, 19]. Pathogenic variants within 10 other mt‐tRNA genes [20, 21, 22, 23, 24, 25, 26, 27, 28, 29, 30, 31, 32] and variants in genes encoding mtDNA‐encoded structural subunits of complex I, complex III and complex IV also cause MELAS [33, 34, 35, 36, 37, 38, 39, 40, 41]. A MELAS‐like phenotype associated with mutations in the nuclear gene encoding the catalytic subunit of the mitochondrial DNA polymerase gamma (*POLG)* has also been reported [42].

Despite its initial identification in patients with MELAS, only 15–40% of patients clinically diagnosed as carriers of m.3243A>G have a MELAS diagnosis [43, 44]. The most common syndrome associated with m.3243A>G is MIDD (maternally inherited diabetes and deafness) [45], but PEO (progressive external ophthalmoplegia) [46] and, more rarely, MERRF [47] and Leigh syndrome (a progressive subacute‐necrotising encephalomyelopathy, often presenting within the first months or years of life) [48] are also associated. However, classifying patients by these classic syndromes does not fully describe the wide phenotypic spectrum; many patients exhibit overlap syndromes, some display phenotypes that do not fit a syndromic diagnosis, and others are asymptomatic [43].

**Fig. 2:**
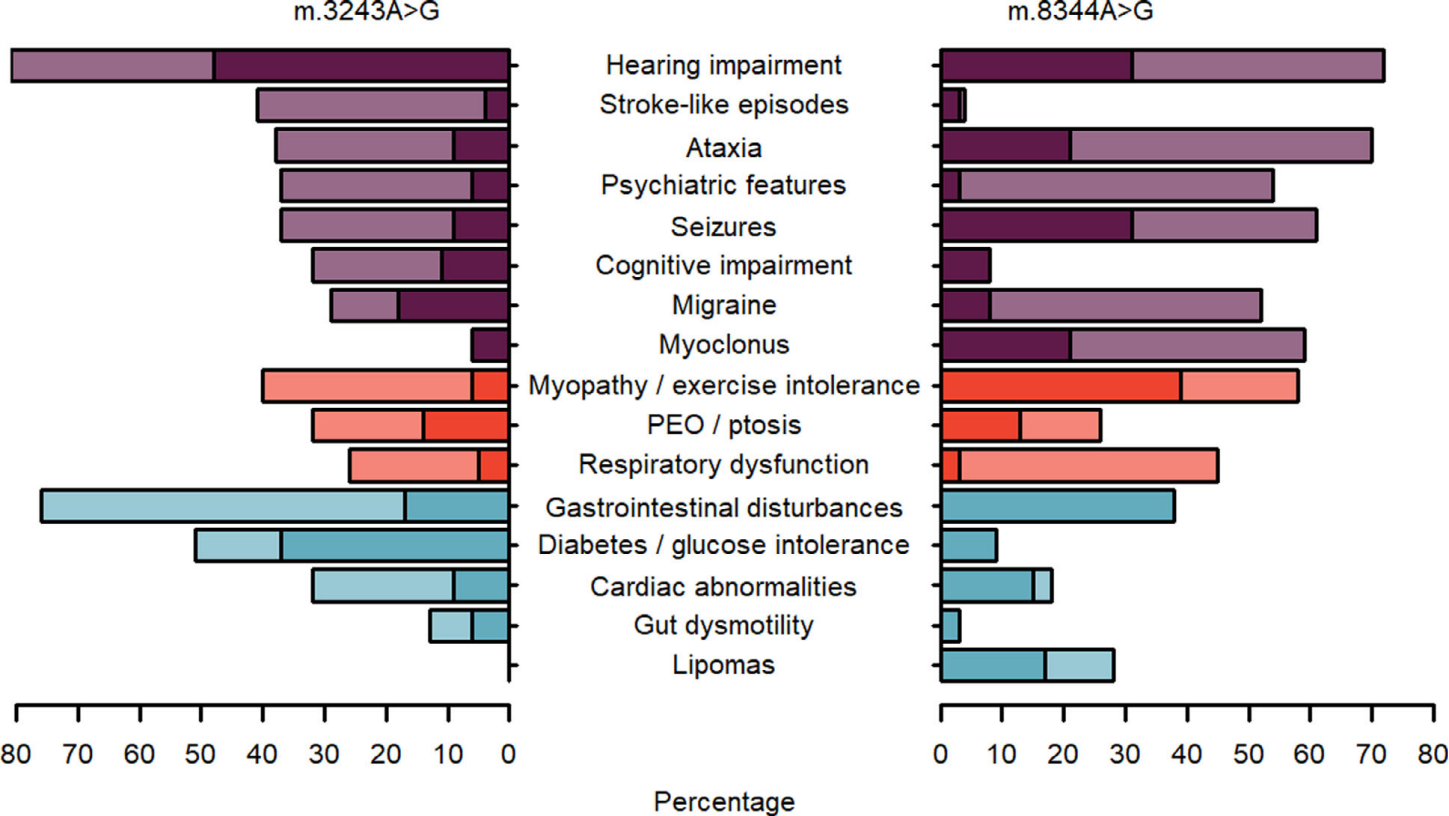
Comparison of the phenotypic features of m.3243A>G and m.8344A>G‐related disease. For m.8344A>G, phenotypic frequencies (at last evaluation) were obtained from Refs [95, 97]. For m.3243A>G, frequencies were obtained from Refs [44, 50, 51, 52, 65]. Phenotypes are grouped into neurological (purple), muscle‐related (red) and others (light blue) and ordered by frequency in m.3243A>G patients. Stroke‐like episodes, cognitive impairment, diabetes and gut dysmotility are notably higher in frequency in m.3243A>G patients, whereas ataxia, psychiatric features, myoclonus and myopathy/exercise intolerance are higher in m.8344A>G patients. Lipomas are a noticeable feature of m.8344A>G but not m.3243A>G‐related disease. For phenotypes where more than one estimate of frequency is reported in the literature, the highest estimate is shown in a lighter shade, with the lowest estimate in a darker shade, thus depicting an estimated range.

Cohort studies can give us insight into the relative incidence of the large number of clinical phenotypes associated with m.3243A>G and are less susceptible to publication bias than case studies, although differences in ascertainment and diagnostic criteria can make comparisons between studies challenging [43, 44, 49, 50, 51, 52]. Nevertheless, it is clear that hearing loss, gastrointestinal disturbances, diabetes/glucose intolerance, cerebellar ataxia, myopathy/exercise intolerance, cardiac involvement, ptosis and psychiatric involvement are among the most common m.34243A>G‐related phenotypic features (Fig. 2). The reported incidence of stroke‐like episodes in m.3243A>G carriers varies considerably, probably reflecting differing ascertainment and follow‐up strategies; the frequency is 17% in the UK Mitochondrial Disease Patient Cohort and 5% in a Dutch cohort of 34 pedigrees, both of which have actively recruited matrilineal relatives of probands, whereas the Nationwide Italian Collaborative Network of Mitochondrial Diseases cohort reports 40% [44, 50, 52].

Other reported clinical features include cognitive impairment/developmental delay [49], neuropathy [53], PEO [46], retinopathy [54], optic neuropathy [47], dysphonia/dysarthria [52, 55], migraine [56], growth failure/short stature [57] and sudden adult death [58]. Less frequently, m.3243A>G has also been associated with a wide range of additional features, including renal failure [59], neuroendocrine dysfunction [60] and vasculitis [61], further widening the phenotypic spectrum associated with this variant.

Disease related to m.3243A>G is progressive and results in a phenotypic profile that changes over time. This is exemplified in an Italian cohort study (*n* = 126; 6.3% asymptomatic), which reported hearing loss (31%), generalised seizures (18%) and diabetes (18%) as the most common phenotypes at disease onset (mean age of onset in symptomatic patients was 23.4 ± 15.6 years). At last evaluation (36.0 ± 20.7 years), these had all increased in frequency (58%, 37% and 41%, respectively) and ptosis/ophthalmoparesis, migraine, muscle weakness, exercise intolerance and heart disease were also present at a frequency > 30% [44]. Progressive disease in patients with MELAS and their carrier relatives has also been reported in a prospective cohort study of American patients [49]. Significant associations between some traits hint towards a similar underlying aetiology or a contemporaneous trigger in already vulnerable tissues, for example seizures, encephalopathy and stroke‐like episodes; diabetes and deafness; cognitive impairment with a number of CNS traits (e.g. stroke‐like episodes, encephalopathy, cerebellar ataxia and dysphonia/dysarthria); and cerebellar ataxia with a number of traits (e.g. myopathy, hearing impairment and dysphonia/dysarthria) [52].

These headline figures give us an indication of the broad range of phenotypic features associated with m.3243A>G, but many show a wide range of severities and are often progressive; for example, ‘gastrointestinal disturbances’ could be characterised by episodes of irritable bowel and/or constipation but can also indicate that a patient has experienced severe gastrointestinal pseudo‐obstruction (paralytic ileus; ~ 10% patients), which can be fatal in the situation of concomitant stroke‐like episodes [62]. Similarly, psychiatric involvement is observed in ~ 70% and can often be mild and transient (e.g. a short period of depression), but in ~ 20–40%, it can be more severe, warranting specialist treatment (e.g. bipolar disorder, psychosis, self‐harm or suicide attempts), and may be linked to cerebellar atrophy [52, 63, 64, 65]. Clinically validated scores such as the Newcastle Mitochondrial Disease Adult Scale (NMDAS) and the Newcastle Paediatric Mitochondrial Disease Scale (NPMDS) can be used to record both the presence and severity of phenotypes, aiding more direct comparisons between cohorts [66, 67].

### Clinical phenotypes of m.8344A>G

The involvement of mitochondrial dysfunction in a syndrome characterised by myoclonus (quick, involuntary skeletal muscle jerks), and epilepsy was reported in two unrelated patients by Fukuhara *et al*. [68] in 1980, and termed MERRF (myoclonic epilepsy associated with ragged‐red fibres). Ragged‐red fibres (RRFs), so named due to their excessively red appearance when stained with Gomori's trichrome and the ragged outer edge of the fibre caused by the accumulation of abnormal subsarcolemmal mitochondria, are histopathological hallmarks of mitochondrial disease observed in patient muscle biopsies [68, 69]. A decade later, after Wallace *et al*. [70] had demonstrated the presence of a maternally inherited mtDNA variant, a heteroplasmic A‐to‐G transition at position 8344 in the mitochondrial genome within *MT‐TK* was identified as the cause of MERRF syndrome [71], a finding that was corroborated later that year [72]; further evidence suggested that it was pathogenic, causing abnormal mitochondrial translation, and was shown to have arisen independently in three different individuals with MERRF [73]. Other variants within *MT‐TK* have also been associated with the MERRF phenotype, for example m.8356T>C and m.8363G>A [74, 75], as well as variants in five other mt‐tRNA genes [21, 27, 47, 76, 77] and *MT‐ND5* [78], but m.8344A>G remains the most common pathogenic variant associated with the MERRF phenotype [10].

In the 30 years since the identification of m.8344A>G, over 350 additional mutation carriers have been reported in the literature and the phenotypic spectrum associated with this pathogenic variant has expanded from the canonical phenotypes of myoclonus, seizures, cerebellar ataxia and mitochondrial myopathy to include many others such as hearing impairment [71], lipomas [79], ptosis and ophthalmoparesis [80], cardiac abnormalities [81, 82], respiratory impairment with sleep disorders [83] and diabetes [84] (Fig. 2).

Less commonly, m.8344A>G has been reported to cause a severe childhood presentation of Leigh syndrome, global developmental delay, hypotonia, ataxia, dystonia and ophthalmologic abnormalities [46, 85, 86, 87, 88, 89]. m.8344A>G is also the cause of the independently described Ekbom's syndrome (photomyoclonus, cerebellar ataxia and cervical lipoma) [90]. Stroke‐like episodes and gastrointestinal dysfunction (paralytic ileus), both more commonly associated with the m.3243A>G variant, have also been linked to m.8344A>G [91]. Distal weakness with respiratory insufficiency was reported in one patient [92] and Parkinsonism in another [93], although m.8344A>G is not a common cause of Parkinsonism [94]. Although many of these studies have not investigated the possibility of the presence of mtDNA *cis*‐modifiers, some report familial studies in which other maternal relatives do not exhibit such severe clinical manifestations, suggesting that something else may be modulating the phenotype in these individuals [85, 88]. However, maternal inheritance of more severe phenotypes has been observed in some pedigrees, which might indicate the presence of *cis*‐modifiers [86, 87].

As m.8344A>G is less frequent than m.3243A>G, fewer and smaller cohort studies have been performed, but they still provide insight into the full phenotypic spectrum. Mancuso *et al*. [95] reported phenotype data from 42 individuals carrying m.8344A>G, representing 12.4% of all patients with pathogenic mtDNA variants in the Italian Collaborative Network of Mitochondrial Diseases. The mean age of onset was 30.1 ± 18.4 years (range 0–66), although they observed childhood onset (< 16 years) in 35.7% and about 10% were asymptomatic. Signs of neuromuscular dysfunction were present in 76.5% of symptomatic adult patients, consistent with a previous study [96]; muscle weakness (58.8%), exercise intolerance (44.1%), generalised seizures (35.3%), hearing loss (35.3%) and multiple lipomas (32.4%) were also common phenotypes (> 30%). By combining clinical data from their 42 individuals with that of 282 cases available in the literature, Mancuso *et al*. estimated that the most common phenotypes across all reported carriers were myoclonus, muscle weakness and ataxia (35–45%), followed by generalised seizures and hearing loss (25–34.9%), then cognitive impairment, multiple lipomas, neuropathy and exercise intolerance (15–24.9%). The least common were ptosis/ophthalmoparesis, optic atrophy, cardiomyopathy, muscle wasting, respiratory impairment, diabetes, muscle pain, tremor and migraine (5–14.9%) [95].

A more recent cohort study from the German network for mitochondrial disorders (mitoNET) reported a slightly lower age of onset (24.5 ± 10.9 years; range 6–48) but with a similar rate of childhood onset (25%) [97]. These authors reported that a larger proportion of patients had myoclonus, muscle weakness, ataxia, seizures and hearing loss (58–72%), respiratory impairment, increased CK, migraine and psychiatric involvement (45–54%) and gastrointestinal dysmotility and swallowing impairment (35–38%). However, the proportion of patients with RRFs was lower (63% vs. 96%). Interestingly, another small cohort study (*n* = 15) also reported a much higher proportion of patients with respiratory impairment (67%), with nearly 50% of patients requiring either nocturnal ventilator support or tracheostomy with mechanical ventilation [83]. Different ascertainment methodologies may account for some of the discrepancies in prevalence data; for example, the German study excluded 22 symptomatic family members who had not been genetically confirmed to carry m.8344A>G [97]; however, it is clear from these cohort studies that the phenotypic spectrum is wide and multisystemic and can overlap with phenotypes associated with other pathogenic mtDNA variants, such as m.3243A>G (e.g. stroke‐like episodes).

Both m.3243A>G and m.8344A>G present with wide, overlapping clinical spectra. Consequently, identifying the underlying cause of a patient's disease is frequently dependent upon obtaining a molecular diagnosis. Although lipomas are more commonly associated with m.8344A>G, and a family history of deafness and diabetes might point towards m.3243A>G (Fig. 2), phenotypic features are diverse and most patients, especially those with m.3243A>G, do not fit a syndromic diagnosis. Therefore, in the diagnostic setting, patients who are suspected of carrying a pathogenic mtDNA variant will be screened for a number of common variants, m.3243A>G and m.8344A>G among them [98].

Currently, our understanding of the phenotypic spectrum associated with both m.3243A>G and m.8344A>G is biased towards individuals who have been referred for genetic testing, that is patients showing symptoms linked to mitochondrial disease and their relatives. However, an estimated 140–250 in 100 000 individuals carry the m.3243A>G variant [99, 100], which is 40–70 times higher than the estimated disease prevalence of 3.5 in 100 000 [10]. This implies that the proportion of asymptomatic carriers is high. Disease prevalence (0.2 in 100 000) is far lower for m.8344A>G, and we lack a good estimate of carrier frequency, but asymptomatic or mildly affected carriers are also likely to be present in the general population [10, 100]. Recent developments in the collection of large population‐based genetic and phenotypic datasets, such as the Genomics England's 100 000 Genomes Project and the UK Biobank [101, 102], may allow us to begin to build a more comprehensive picture of the phenotypic spectrum at a population level, although the continued longitudinal and thorough phenotype profiling that is performed within mitochondrial disease clinics and research environments will continue to deepen our knowledge of the phenotypes associated with these highly heterogeneous disorders. This will be essential if we are to gain a deeper understanding of the additional factors associated with an increased likelihood of severe disease in individuals who carry m.3243A>G or m.8344A>G.

### Heteroplasmy and mtDNA copy number, disease burden and progression

Both m.3243A>G and m.8344A>G are heteroplasmic variants (Box 1). It is very tempting therefore to hypothesise that the vast clinical heterogeneity observed in individuals carrying these mt‐tRNA variants could be attributed to differences in levels of the variant allele between cells, tissues and individuals. In reality, the relationship between pathogenic mt‐tRNA levels and clinical phenotype is not straightforward.

Both m.3243A>G and m.8344A>G are functionally recessive *in vitro*; low levels (6–15%) of wild‐type mtDNA are capable of rescuing mitochondrial translation, complex IV activity and oxygen consumption to near‐normal levels [103, 104]. However, the picture is more complicated *in vivo*; high levels of m.3243A>G have a dominant‐negative effect in skeletal muscle fibres, impeding rescue of the respiratory chain defect by increased wild‐type mtDNA copy number [105]. For both variants, mutation‐associated phenotypes are observed in some patients who harbour low levels of mutated mtDNA, whereas some individuals with high mutation levels only manifest mild symptoms [97, 106, 107].

With regard to m.3243A>G, deciphering the relationship between the level of the pathogenic (G) allele and disease burden or clinical phenotype is further complicated by an age‐related negative selection against this G allele in easily sampled mitotic tissues, such as the blood [108, 109, 110, 111]; in contrast, levels in postmitotic tissues such as the muscle appear to be more stable [106]. Blood levels can be corrected for age to more accurately reflect the likely levels of m.3243A>G within the postmitotic tissues most affected by the mitochondrial dysfunction. Indeed, despite interindividual variation in the rate of this age‐related decline, age‐corrected blood m.3243A>G levels are better correlated with total disease burden and progression than urine levels and are just as good as muscle levels in this respect [106]. Despite this, models using age and age‐corrected m.3243A>G levels can only explain ~ 27% of the variability in disease burden that we observe. Increased muscle mtDNA copy number has some protective effect (increasing the explained variation to ~ 40%), implying that a higher wild‐type mtDNA content may rescue mitochondrial dysfunction, but the lower mtDNA copy number in more severely affected individuals may simply reflect deconditioning of skeletal muscle due to decreased levels of physical activity within these patients [106, 112].

The relatively high frequency of m.3243A>G has allowed the relationship between variant level and phenotype to be explored in large cohorts. The first of these studies reported higher frequencies of severe neurological phenotypes, such as stroke‐like episodes, epilepsy and ataxia, in patients with higher m.3243A>G levels, but the opposite was the case for features such as CPEO, myopathy and deafness; this apparent paradox may result from earlier presentations in individuals with neurological symptoms and highlights the need to also consider age when studying progressive disorders [96]. Later studies failed to use m.3243A>G levels to predict specific phenotypes, although patients with MELAS tend to have higher levels compared with their carrier relatives [44, 49, 51]. Most recently, in a cohort study of 238 adult carriers of m.3243A>G, increasing mutation levels were significantly associated with increasing severity of cerebellar ataxia, cognition, neuropathy, dysphonia–dysarthria, seizures, encephalopathy, stroke‐like episodes, hearing impairment, myopathy, diabetes and cardiovascular involvement; cerebellar ataxia, neuropathy, hearing, myopathy, diabetes and cardiovascular involvement were more severe with increasing age [52]. Both migraine and seizures were found to be more severe in younger individuals, likely reflecting earlier presentations in these individuals. Hearing impairment, diabetes and cerebellar ataxia were the traits best explained by mutation level and age, but only a small proportion of the variability in all phenotypes was explained by these factors (pseudo‐*R* ^2^ values ranged from 0.02 to 0.17). Therefore, much of the phenotypic variability we see in m.3243A>G‐related disease remains to be explained.

In the case of m.8344A>G, an early attempt to correlate levels of the variant allele in muscle with clinical phenotypes in 55 individuals (including cases from both a clinical cohort and the literature) showed that the frequency of myoclonus, ataxia, epilepsy, myopathy, deafness and dementia was higher in individuals with high levels. Of note, this cohort included only two individuals with levels < 70%, possibly reflecting a publication bias [96]. Two more recent studies have failed to see significant relationships between mutation level and clinical phenotype, although a nonsignificant trend towards higher levels in patients with myopathy was observed in the Italian cohort [95, 97]. However, conclusions are compromised by relatively low statistical power (sample sizes were 22 and 18). Thus, understanding the relationship between m.8344A>G level and phenotype will require the analysis of larger patient cohorts.

### Segregation and clonal expansion

The phenomenon of heteroplasmy results in variable levels of pathogenic mtDNAs being present in different cells, tissues and individuals (Box 1) [108, 113, 114, 115]. Moreover, the levels of m.8344A>G and m.3243A>G are higher in cells that show defective OXPHOS compared with biochemically normal cells from the same tissue [116, 117, 118, 119, 120]. The level of mutation over which a cell manifests OXPHOS deficiency (threshold level of mutation) also differs between individuals, therefore, it is possible that interindividual differences in this threshold, as well as the cellular distribution of mutation levels, could account for some of the clinical variability associated with mtDNA mutations [120].

The cause of these intraindividual differences in mutation levels is not yet fully understood but is likely to be linked to several processes. These include the following: differences in the rate of replication of genomes containing wild‐type and mutant alleles; differential random segregation of mtDNA molecules into daughter cells at cell division; and differences in survival and replication rates of cells with differing mutation levels. During embryo‐fetal development, mutation levels appear to be similar across different tissues; therefore, variability of mutation level between tissues is likely to take place after this early developmental phase [121]; whether there is variability between single cells at this stage is unknown. The control of mtDNA replication can be linked to the cell cycle (‘strict replication’), but the mitochondrial genome is also able to replicate independently of the cell cycle in a process termed ‘relaxed replication’ [122, 123], which may also contribute to clonal expansion and therefore cell‐to‐cell variability, even within postmitotic tissues. Simulation studies show that the clonal expansion of mtDNA point mutations can be explained by the process of differential segregation due to random drift [124] and extensively reviewed in [125]. However, nuclear control of tissue‐specific mtDNA segregation has been demonstrated in mice and similar patterns of m.3243A>G tissue segregation have been described in two sets of identical twins, suggesting that this process may not be fully random [126, 127].

## Pathogenicity of the m.8344A>G mt‐tRNA^Lys^ and m.3243A>G mt‐tRNA^Leu(UUR)^ variants

The development of therapies for mitochondrial disease is a rapidly evolving area, with efforts focussed on discovering small molecules that can improve OXPHOS function, developing potent antioxidant molecules, delivering therapies that can reduce the proportion of mutant mtDNA in affected tissues and preventing the transmission of pathogenic mtDNA variants (reviewed extensively in [128]). To develop effective therapies for patients with mitochondrial disease, a complete understanding of the specific molecular mechanisms and key factors that sustain mtDNA expression is essential. Mitochondrial diseases are clinically heterogeneous, and some pathogenic variants display striking tissue specificity (e.g. lipomas associated with m.8433A>G and retinal nerve atrophy associated with variants in mitochondrially encoded complex I genes) [129]. In stark contrast to this complex clinical picture, the current prevailing view on the aetiology of these clinical features is that they are caused by OXPHOS deficiency. General aberrations in mitochondrial genome expression are likely to generate an OXPHOS defect, but the question remaining then is ‘Why are these mitochondrial diseases so heterogeneous?’ We and others have proposed that this is due to variation in the underlying molecular mechanism for each mtDNA mutation, but addressing this question experimentally is complicated by differences in heteroplasmy (see Box 1) across specific cell types and tissues in patients.

Pathogenic variants in mt‐tRNA genes can destabilise the mt‐tRNA molecule, prevent efficient or correct aminoacylation and/or affect the interaction with elongation factor Ef‐Tu and the ribosome. As a consequence, mitochondrial protein synthesis is perturbed. Hence, steady‐state protein levels of the 13 mtDNA‐encoded proteins are reduced resulting in diminished respiratory chain complex abundance. While the frequencies of UUR (Leu) and AAR (Lys) codons are similar in human mtDNA‐encoded proteins, some complexes are preferentially affected depending on the frequency of the encoded amino acid in the specific subunit [130]. Thus, respiratory chain dysfunction clearly is a direct consequence of the primary molecular defect, and redox state and energy‐generating capacity of the affected cells and tissues are compromised. The level of the variant allele in patient tissues is clearly one major factor that can determine disease severity and progression; however, as discussed above, for m.3243A>G and m.8344A>G, variant levels are not strictly correlated with disease severity, age of onset or the rate of progression. For m.3243A>G, levels of the variant allele diminish over time in mitotic tissues; the molecular mechanism of this is not fully understood but is likely to be related to cell division (Box 1) [111]. Mitochondrial translation‐dependent proliferation defects have been attributed to deficient aerobic energy metabolism, even though proliferating cells rely primarily on glycolysis to meet their metabolic demands. Studies using small molecule inhibitors of mitochondrial translation have indicated that a defect in cell proliferation can occur before OXPHOS is impaired and it has been speculated that the health of mitochondrial translation itself might be an important checkpoint for cell cycle progression [131, 132, 133].

The demand for OXPHOS is very high after birth, indicating the prevalence of glycolytic metabolism during fetal development and indispensability of OXPHOS for postnatal maintenance of human tissues [134]. Therefore, significant loss of respiratory chain capacity offers a consistent rationale for the rapidly progressing disease onset soon after birth, with organ failure and lactic acidosis. However, even with relatively high variant levels of m.3243A>G or m.8344A>G and concomitant loss of respiratory chain complexes, a great number of patients develop progressive disorders only later in life. This indicates that patient‐specific modifiers exist and that specific organs have distinct age‐dependent sensitivity to respiratory chain dysfunction. It is also in line with studies on the coevolution of mtDNA‐encoded tRNAs and their cognate mt‐aaRSs [7] and direct evidence for the existence of nuclear modifiers comes from the observation that overexpression of human mt‐leucyl‐tRNA synthetase is sufficient to improve the viability of m.3243A>G cells when grown in galactose [135]. Interestingly, overexpression of the carboxy‐terminal domain alone was both necessary and sufficient for the rescue [136]. The hypothesis that mt‐aaRSs have the capacity to modify disease outcomes in m.3243A>G warrants the screening for disease‐modifying SNPs of the cognate mt‐aaRSs in a large cohort of m.3243A>G patients.

## Molecular mechanisms

In recent years, tremendous progress has been made in deciphering the primary insults causing mitochondrial gene expression disease. Avoiding the limitations of studying pathogenic mtDNA variants in *trans*mitochondrial cybrids (cybrids), Shoubridge *et al*. selected clones of patient myoblasts homoplasmic for m.3243A>G and m.8344A>G, studying control cells, homoplasmic for wild‐type mtDNA, from the same patient. This tour de force established cell culture model systems to investigate homoplasmic mutant cells and use wild‐type mtDNA cells with the same nuclear genetic background as controls for both m.8344A>G and m.3243A>G patient myoblasts [103, 130]. These cell lines have been extremely valuable in isolating the primary molecular aberrations and their downstream consequences for both m.8344A>G and m.3243A>G. Because mitochondrial gene expression is dependent on two interacting genomes, these myoblasts provide the gold standard, avoiding the use of cell models with unstable and transformed nuclear backgrounds. Unlike HEK293 cells, the m.8344A>G patient myoblasts closely recapitulate the molecular findings from patient muscle samples [137]. Studies of the molecular mechanisms underlying both m.8344A>G and m.3243A>G should make use of this study system.

The pathogenic m.3243A>G variant causes impaired recognition of UUA and UUG codons but only minor aberrations in translation elongation rates; amino acid misincorporation consequently induces the decreased stability of *de novo*‐synthesised mtDNA‐encoded proteins [103, 130]. mt‐tRNA molecules require a wide variety of post‐transcriptional modifications, which stabilise mt‐tRNA structure, enable efficient interaction with the ribosome and ‘fine‐tune’ mitochondrial translation (Fig. 1B). In m.3243A>G, the taurine modification at the wobble position in mt‐tRNA^Leu(UUR)^ is disrupted, which in turn causes the mt‐tRNA to decode all UUX codons [138]. Thus, misincorporation as the primary molecular phenotype ultimately causes lack of respiratory subunits and OXPHOS deficiency. Importantly, it also provokes a burden on the inner membrane proteostasis machinery dealing with unstable respiratory complex complex intermediates and very hydrophobic, polytopic and *de novo*‐synthesised respiratory chain subunits [139]. These proteins are associated with iron sulfur clusters and haem molecules already inserted into the inner membrane and need to be recognised and extracted from the membrane by chaperones. Subsequently, degradation by dedicated proteases has to occur. Considering the electrochemical charge across the inner mitochondrial membrane, this quality control system for newly synthesised respiratory chain subunits is especially challenging for the cell.

More recently, a second modification 2‐methylthiolation (ms^2^) was also found to be decreased in m.3243A>G cells [140]. However, since the mt‐tRNA^Leu(UUR)^ is not ms^2^ modified itself – the modification is reduced on mt‐tRNAs decoding Phe, Tyr, Trp and Ser codons – this argues for feedback regulatory mechanisms adapting the modification status of mt‐tRNAs in response to aberrant mitochondrial translation. This is further substantiated by the observed correlation of heteroplasmy level with the ms^2^ modification level in patients [140] and raises the question of which signals transmit and induce this stress response. While reactive oxygen species have been proposed to be the causative entity [140], a more general interpretation entirely consistent with the current data might be that redox changes are involved in signalling the health status of mitochondrial translation. Taken together, the analysis of the primary molecular aberration predicts that the m.3243A>G variant produces both loss and gain of function phenotypes. This is in line with the above‐described patient phenotypes associated with m.3243A>G, where patients with low levels of the variant allele develop disease, and no clear threshold for expression of the mutation is observed in some tissues [107, 141].

Another recent study has suggested that heteroplasmic cells and tissues generated through induced pluripotent stem cell (iPSC) reprogramming technology could be an excellent tool to study tissue‐specific manifestations and mechanisms of pathogenesis in m.3243A>G [142]. The same study shows the mtDNA bottleneck also occurs during reprogramming of m.3243A>G iPSCs, making it a very useful tool to generate and study the impact of varying levels of the pathogenic variant *in vitro*. Furthermore, the authors provide evidence that early‐differentiating neurons show loss of respiratory complex I. Directly measuring protein turnover rates of the respiratory chain subunits from all five complexes would greatly support the claim of active complex 1 degradation in the early neurons. In the light of the heterogeneity of tissue‐specific respiratory chain complex deficiency patterns, insight into a mechanism that actively degrades specific complexes would be a tremendous step forward in our understanding of in m.3243A>G tissue‐specific disease manifestation and allow us to identify the tissue‐specific factors and machinery involved in this process *in vitro*.

In stark contrast to m.3243A>G, the m.8344A>G mt‐tRNA^Lys^ variant causes a very severe translation defect during mitochondrial protein synthesis, readily observable by ^35^S‐metabolic labelling [103, 130, 137]. Decreased steady‐state abundance of mt‐tRNA^Lys^, reduced aminoacylation of the mt‐tRNA, and lack of a post‐transcriptional RNA modification of the anticodon wobble base [143, 144, 145, 146] have all been correlated with the aberrant translation pattern. However, the pattern observed from metabolic labelling of mitochondrial proteins was not consistent with random premature termination at lysine codons. Instead, in cells homoplasmic for m.8344A>G, distinct aberrantly sized and full‐length polypeptides have been observed [130, 137]. Pulse and pulse–chase metabolic labelling experiments clearly demonstrate that the aberrantly sized chains are unstable, but interestingly, all full‐length nascent chains are also unstable [103, 130, 137]. Reports with contradicting results have been published, but all these studies have been based on cybrids or *in vitro* translation experiments, both of which are not suited to closely recapitulate mitochondrial protein synthesis in tissues of MERRF patients [137].

Thus, even though m.3243A>G and m.8344A>G both decrease the steady‐state abundance of the assembled respiratory chain complexes and ATP synthase, they have different primary molecular aberrations, which may have cellular implications beyond OXPHOS. Different processes are therefore required to resolve insults from these specific primary aberrations in mitochondrial protein synthesis, and it is these processes that are likely to determine the molecular, if not the clinical, phenotype. Additionally, tissue‐ and cell type‐specific stress responses are likely serving as modifiers of mitochondrial disease phenotypes. While aimed to rectify the initial insult by the mt‐tRNA mutation, such stress responses can get deleterious, especially when they become chronic. Both m.3243A>G and m.8344A>G patients show the induction of the mitochondrial integrated stress response ISR^mt^ with high circulating serum levels of FGF21 and GDF15 [147]. Importantly, in mice, inhibition of the chronic ISR^mt^ has been shown to be protective [148].

A more detailed understanding of the specific factors required for the quality control processes is needed and could cast light on the molecular mechanism behind the different tissue specificities (and therefore clinical manifestations) of m.3243A>G and m.8344A>G. Recently, a methodological advance in next‐generation RNA sequencing allowed Richter *et al*. to investigate the consequences of homoplasmic m.8344A>G variant levels on the stoichiometry and modifications of all mt‐tRNAs in affected patient tissues and in the above‐described homoplasmic m.8344A>G myoblasts. Contrasting the results in HEK293T cells and cybrids, homoplasmic m.8344A>G myoblasts faithfully represented the molecular muscle phenotype with steady‐state level and wobble base modification specifically reduced on mt‐tRNA^Lys^ only [137].

The results also uncovered a prevalent and unsuspected influence of RNA modifications on mitochondrial gene expression in general and revealed the role of a second mt‐tRNA methyl modification at position A8348 (besides the wobble base taurine modification) in the pathogenesis of MERRF. The mutant mt‐tRNA appears largely unable to efficiently decode lysine during translation elongation, and in contrast to the m.3243A>G mutation, elongation rates are severely reduced in m.8344 A>G myoblasts [103, 130, 137]. The impact of the second mt‐tRNA methyl modification at position A8348 was revealed by overexpression of the writer enzyme TMRM61B (Fig. 1B). While re‐establishing the modification to control levels rescued the translation elongation phenotype, all translation products were still unstable, mimicking the molecular translation phenotype observed in m.3243A>G myoblasts. Conversely, overexpressing the TMO1 part of the writer enzyme complex of the mnm5s2U34 wobble uridine base rescued both the elongation rate and stability of all mitochondrial translation products [137]. Because MTO1 activity is directly correlated with mitochondrial translation fidelity, enhancing fidelity but not translation initiation or elongation rate would be an attractive therapeutic avenue in both m.3243A>G and m.8344A>G‐related mitochondrial disease. Enhanced fidelity is advantageous when compared with enhancing translation *per se*, because it prevents mitochondrial translation quality control systems from getting overwhelmed [5].

Together, these findings strongly suggest that the expression levels of mt‐tRNA modification factors could be modifiers of mt‐tRNA‐associated disease and partly explain the highly variable tissue‐dependent phenotypes. The very specific molecular aberrations observed in m.8344A>G are also in line with the view that the mutation causes a strong burden on ribosome quality control where severe stalling of the mitochondrial ribosomes during translation elongation occurs. Rescuing ribosomes that fail to synthesise full‐length polypeptides requires a rescue pathway to recover the stalled ribosomes, including endo‐ and exoribonuclease activity to cleave the mRNA, a release factor to catalyse the cleavage of the ester bond of the peptidyl‐mt‐tRNA, and chaperones and proteases to recognise and degrade the premature translation product. It is therefore unsurprising that pathogenic variants in mt‐tRNAs present as a heterogeneous class of diseases with varying severity and variable tissue specificity. We are just starting to understand the multiple layers and factors involved in the modification of molecular insults from both mutations; discovering more modifying factors by utilising the ever‐expanding genetic tool kit, and importantly primary patient material, will likely be a very fruitful avenue to pursue in the near future. For both m.3243A>G and m.8344A>G, we postulate that the molecular mechanisms driving pathology are multilayered, consisting of a combination of multiple patient‐ and tissue‐dependent factors from mt‐tRNA stoichiometry, modification, aminoacylation and their quality control through to translation elongation speed and fidelity. Disease manifestation will also be affected by nascent chain handling and proteolytic quality control of the 13 mtDNA‐encoded proteins and by cellular stress responses that not only modify the coordination of the gene expression from both nuclear and mitochondrial genomes but also drive metabolic changes in the patients.

## Mito‐nuclear crosstalk

Current estimates place ~ 1500 proteins within the mitochondrial proteome, and with only 13 of these encoded by the mitochondrial genome, mito‐nuclear communication is key to maintaining optimal mitochondrial function and determining how a cell responds to metabolic challenges. Indeed, mitochondria are critically dependent on the coordination of genetic information from both the nuclear and mitochondrial genomes [133, 149, 150, 151, 152]. Characterisation of the communication between these two organelles has revealed a complex and sophisticated bidirectional system involving both anterograde (nuclear to mitochondrial) and retrograde (mitochondrial to nuclear) signalling pathways. We are only beginning to recognise the importance of these communication networks and how they are deployed in mitochondrial diseases.

It has been demonstrated that small changes in m.3243A>G variant level in cybrids can cause large shifts in cellular phenotype and gene expression profiles, despite much smaller effects on oxidative capacity, indicating retrograde signalling and demonstrating the capacity of the nucleus to respond to mitochondrial changes related to mtDNA mutations [52, 153]. NAD+/NADH and α‐ketoglutarate/succinate ratios have been shown to mediate some of these transcriptional changes [154]; these experiments confirm that the presence of pathogenic mt‐tRNA variants at different levels can bring about epigenetic and transcriptional changes and that some of these are mediated by changes in the metabolic status of the mitochondria.

The rewiring of cellular metabolism and regulation of nuclear and mitochondrial transcription in response to mitochondrial dysfunction involves initiation of the ISR^mt^, but we are only just beginning to understand how this response is triggered. Recently, Fessler *et al*. identified a pathway that may constitute a missing piece in this puzzle, demonstrating that the stress‐induced activation of OMA1, an inner membrane mitochondrial protease, leads to the cleavage of DELE1 and its accumulation in the cytosol; subsequent binding of this shortened form of DELE1 to HRI (an eIF2ɑ kinase) triggers the expression of the transcriptional regulator C/EBP homologous protein (CHOP), a key component of the ISR^mt^, which is induced upon misfolding of proteins in the mitochondrial matrix and aberrations in mitochondrial gene expression [148, 155, 156, 157]. It is now crucial to study the impact and dynamics of this pathway in response to the presence of both m.3243A>G and m.8344A>G to understand its importance in health and disease, more specifically in aberrations where mitochondrial protein synthesis is disrupted either by ribosomal stalling or by amino acid misincorporation.

Very recently, digenic inheritance of mtDNA and nuclear variants has been demonstrated for reversible infantile respiratory chain deficiency, which is associated with the homoplasmic m.14674T>C mt‐tRNA^Glu^ variant, but shows reduced penetrance [158, 159]. In affected patients, Hathazi *et al*. discovered additional rare, heterozygous nuclear mutations in *EARS2* and *TRMU*, which encode proteins responsible for aminoacylation of mt‐tRNA^Glu^ and mt‐tRNA^Gln^ and cysteine‐dependent thiouridylation of mt‐tRNA^Glu^, as well as other genes involved in glutamate or glutamine metabolism. Studying the transcriptome and proteome within skeletal muscle from these patients, who undergo spontaneous recovery by 5–20 months of age, revealed that the ISR^mt^ is triggered in early life (characterised by increased FGF21 and GDF15 expression), and is followed by an increase in metabolism modulated by serine biosynthesis, one‐carbon metabolism, lipid oxidation within the TCA cycle and amino acid availability. The authors hypothesise that this subsequently leads to mTOR‐dependent increased mitochondrial biogenesis and recovery from the respiratory chain deficiency [159]. Given the protective effect of increased mtDNA copy number in muscle that we see in m.3243A>G‐related disease [106] and the increased FGF21 and GDF15 expression observed in patients with MERRF and MELAS [147], it is possible that a similar mechanism is operating in response to other pathogenic mt‐tRNA variants. Interindividual variation in the effectiveness of this response may contribute to differences in disease burden between patients with similar levels of mutation. However, unlike m.14674T>C, m.3243A>G and m.8344A>G are heteroplasmic variants and the disease they cause is more heterogeneous and not reversible; teasing out the factors that affect clinical presentation is going to be hugely challenging.

## Future directions

We cannot explain and do not understand what causes the majority of the phenotypic variability in patients carrying m.3243A>G and m.8344A>G. The levels of mutated mtDNA and patient's age are thought to be involved but variation within nuclear genes is also a strong contender. Recent pedigree analyses suggest that additive genetic factors play a considerable role in the severity of a number of m.3243A>G‐related phenotypes. Moderate to high heritability estimates were obtained for psychiatric involvement, hearing impairment, ataxia, migraine and cognition [52]. Interestingly, the presence of encephalopathy was also shown to be heritable. Yet, high levels of m.3243A>G alone are not sufficient to explain the presence of the most severe and life‐limiting phenotypes associated with this mtDNA variant. Although similar studies have not yet been performed for m.8344A>G, the lack of strong association of phenotypes with the proportion of the variant allele suggests that variation within nuclear genes may also contribute to the wide clinical heterogeneity associated with this variant. Indeed, there may be some overlap. Identifying these nuclear variants will give us more clues as to the molecular mechanisms underlying the pathogenicity of mt‐tRNA variants, identify potential therapeutic targets and could improve genetic counselling.

To this end, two complementary approaches can be employed to identify additional nuclear factors that are key to understanding the molecular mechanism and hence the phenotypic expression of these mt‐tRNA variants. The first is hypothesis‐driven, building on the knowledge we already have of the molecular mechanisms of pathogenicity and molecules involved in mito‐nuclear crosstalk to identify further interacting molecules in these pathways. This approach has already been successful in a mouse model of neurodegeneration caused by aberrant mitochondrial inner membrane structure [160] and in the nonstop *MT‐ATP6* mRNA (m.9205delTA) patient cell line [161]. Also, it has been shown previously that in cell lines harbouring ‐tRNA mutations, overexpression of the cognate mt‐aaRSs can suppress the mutant phenotypes [162, 163].

The second is a knowledge‐discovery approach, which is not limited by current knowledge and has the potential to uncover unknown pathways and molecular mechanisms of disease. Combining both approaches will likely prove to be more powerful than either in isolation.

Knowledge‐discovery approaches employ methods such as genetic linkage analysis, genome‐wide genetic association (GWAS) and whole‐genome/exome sequencing to systematically search the nuclear genome for regions and variants that modify phenotype. This has been hugely successful in recent years at identifying nuclear risk factors for a wide range of complex traits; the GWAS catalogue contains over 70 000 variant–trait associations and is constantly growing [164, 165]. Applying these methods to mt‐tRNA diseases requires the collation of large, deeply phenotyped, patient cohorts and pedigrees, requiring strong collaborations between basic scientists and clinicians across the world, and a systematic approach to phenotyping. The rare nature of mtDNA‐related disease will result in smaller cohorts than those typically utilised for common disorders, but genetic variation with large effect sizes should be identifiable, even in modestly sized studies, particularly if information from families is incorporated into the analyses [166]. Whole‐genome sequencing and whole‐exome sequencing are likely to be more effective than GWAS‐based approaches if there is genetic heterogeneity, as is the case with secondary pathogenic nuclear variants necessary for the phenotypic expression of the homoplasmic m.14674T>C variant [159]. More recently, whole‐genome sequencing and whole‐exome sequencing have been applied to large population‐based cohorts, such as the UK Biobank and Genomics England's 100 000 Genomes Project, enabling the identification of further carriers of mtDNA mutations within populations; these may be valuable resources that can be used for validation studies [101, 102].

By using the knowledge gained from identifying the nuclear factors involved in the complex molecular pathology of mt‐tRNA‐related mitochondrial disease, we will be able to inform and direct further hypothesis‐driven studies, characterising the essential pathways and networks responsible for determining cellular fate in response to defects in mitochondrial translation. These advances and elegant patient‐derived cell culture techniques have the capacity to transform our understanding of the molecular pathogenesis and the basis of the extreme clinical heterogeneity that is associated with pathogenic variants in mt‐tRNA genes. Such knowledge will be instrumental to identify potential targets for improved diagnostics and pharmacological treatment for patients with no treatment option to date.
